# Silver Nanoparticles from *Duddingtonia flagrans*: Evaluation of Potential Ovicidal Activity on *Toxocara canis* Eggs

**DOI:** 10.3390/pathogens13121043

**Published:** 2024-11-27

**Authors:** Carolina Magri Ferraz, Lara Coslop Comério, Vinícius Bastos Salles Segantine, João Pedro Barbosa de Assis, Laryssa Pinheiro Costa Silva, Lara De Nadai Rodrigues Bezerra, Jackson Victor de Araújo, Vinícius Longo Ribeiro Vilela, Filippe Elias de Freitas Soares, Gabriel Augusto Marques Rossi, Fernando Luiz Tobias, Helio Langoni, Fabio Ribeiro Braga

**Affiliations:** 1 Laboratório de Parasitologia Experimental e Controle Biológico, Universidade Vila Velha, Rua São Joao, 48, Vila Velha 29101-420, ES, Brazil; laracomerioo@gmail.com (L.C.C.); viniciusbsegantine@gmail.com (V.B.S.S.); jpbarbosa-deassis@hotmail.com (J.P.B.d.A.); laryssa.pinheirocs@gmail.com (L.P.C.S.); laradnadai@hotmail.com (L.D.N.R.B.); gabriel.rossi@uvv.br (G.A.M.R.); tobias@uvv.br (F.L.T.); helio.langoni@unesp.br (H.L.); fabio.braga@uvv.br (F.R.B.); 2 Departamento de Medicina Veterinária, Universidade Federal de Viçosa, Avenida PH Rolfs, Viçosa 36570-000, MG, Brazil; jvictor@ufv.br; 3 Departamento de Medicina Veterinária, Instituto Federal da Paraíba—IFPB, Rua Presidente Tancredo Neves, Sousa 58800-970, PB, Brazil; vilelavlr@yahoo.com.br; 4 Departamento de Química, Universidade Federal de Lavras, Rua Professor Edmir Sá Santos, Lavras 37203-202, MG, Brazil; filippe.soares@ufla.br

**Keywords:** biological control, *Duddingtonia flagrans*, nematophagous fungus, nanomedicine, nanobiotechnology, *Toxocara canis*

## Abstract

The filtrate of the nematophagous fungus *Duddingtonia flagrans* produces silver nanoparticles (AgNPs) with nematicidal potential. However, there are currently no reports of its activity against *Toxocara canis* eggs. The aim of this study was to investigate the potential ovicidal activity of AgNPs–*D. flagrans* on *T. canis* eggs. *T. canis* eggs were obtained from the dissection of the uterus of adult female nematodes. After the biosynthesis of AgNPs, two experimental assays (A and B) were performed. In assay A, the ovicidal activity of AgNPs on eggs was evaluated after 15 and 30 days of interaction. In assay B, the inhibition (development) of the eggs was measured after 30 days of interaction. The results of assay A showed that the AgNPs destroyed an average of 47% of the eggs tested by the end of the experiment, causing significant structural damage. In assay B, an inhibition rate of 88% was observed at the end of 30 days. The results of the ovicidal activity of AgNP–*D. flagrans* were promising and indicate the potential for future studies on these biomolecules with ovicidal properties.

## 1. Introduction

Recent advances in nanotechnology, especially at the nanometer scale and based on the application of biological techniques, have led to a new field known as nanobiotechnology [1]. This field deals with the manipulation of materials and systems on a scale of 1 to 100 nanometers, which includes nanoparticles (NPs) [2,3]. NPs are characterized by their small size and large surface area. In addition, they have been reported to exhibit a broad spectrum of biological activities, such as antiplasmodial, antibacterial, antifungal, antiviral, and anticancer activities [4,5,6,7,8,9,10].

Conventional NP synthesis methods are often based on chemical processes that raise significant environmental and health concerns due to the production of toxic by-products [11]. On the other hand, nanobiotechnology utilizes innovative approaches that can interact with biological systems. Systems such as plants, algae, bacteria, yeasts, and fungi have emerged as sustainable alternatives for the production of NPs through processes known as biosynthesis or green synthesis. These methods have a lower impact on the environment than conventional chemical processes [12,13].

The biosynthesis of NPs based on fungi and yeasts has attracted considerable interest due to their ability to produce metallic NPs by both intracellular and extracellular enzymatic reduction [2,14]. Nematophagous fungi have shown particular advantages in the biosynthesis of NPs [15]. These microorganisms are able to secrete extracellular enzymes. They thrive in nutrient-poor and low-cost culture media and are also able to tolerate the critical laboratory conditions required for large-scale production, such as flow pressure and agitation [4,16].

Over the decades, nematophagous fungi have been proven to be effective in the biological control of gastrointestinal nematodes in domestic animals by trapping and destroying these organisms through mechanical means (sticky traps and constricting nets) or enzymatic activity through the production of hydrolytic enzymes [16,17,18]. Within this group, the genus *Duddingtonia* stands out for its efficacy, especially against nematodes that infect animals and potentially zoonotic species [19]. Commercial biotechnological products containing the species *Duddingtonia flagrans*, such as Bioverm^®^ (Ghenvet, Paulínia, Brazil) and BioWorma^®^ (IAHP, Sydney, Australia), have been successfully used for parasite control in domestic animals under field conditions, showing a promising scale of sustainable biotechnological applications for this fungus [20,21].

In this context, *D. flagrans* utilizes bioactive compounds, enzymatic production, crude enzymatic extracts, commercial products, and nanoparticle (NP) production for this purpose [16,21]. Recent studies have emphasized the biosynthesis, functionality, and potential therapeutic applications of silver NPs derived from *D. flagrans* (AgNPs–*D. flagrans*) [15]. Subsequent research has evidenced that these AgNPs are effective in killing the third-stage (L3) larvae of *Ancylostoma caninum* and strongylid nematodes which are capable of affecting equines and ruminants [22,23,24]. These findings suggest that AgNPs–*D. flagrans* have considerable potential to be used in innovative experimental approaches targeting the different developmental stages of gastrointestinal parasitic helminths. However, metallic NPs’ use in parasitic disease control remains at its early stages, and it highlights the need for further studies to fully explore their promising and innovative applications [25].

The biosynthesis of AgNPs using *D. flagrans* offers significant advantages over chemical methods. This nematophagous fungus not only enables the production of AgNPs with lower environmental impact, due to the absence of toxic reagents, but also secretes extracellular enzymes and bioactive metabolites that can impart unique properties to the nanoparticles. These bioactive compounds may act synergistically with the AgNPs, enhancing their antiparasitic activity, which suggests a distinct efficacy compared to chemically synthesized AgNPs. Furthermore, the *D. flagrans* filtrate contains compounds that exhibit biological activity, potentially augmenting the efficacy of AgNPs derived from this fungus.

The chemical control of potentially zoonotic gastrointestinal nematodes has become a major challenge in recent decades due to factors such as drug resistance, resulting in an increasing number of stray animals in urban areas and environmental contamination becoming infected with the preparasitic forms of these pathogens [26,27]. Among these nematodes, the genus *Toxocara* stands out, causing infections in dogs and cats and, occasionally, in humans [28]. The species *T. canis* and *T. cati* live in the small intestines of dogs and cats, respectively, and can cause severe infections that can be fatal for their hosts. Humans can become accidentally infected by ingesting *Toxocara* spp. eggs found in the environment, resulting in a disease known as toxocariasis [29,30].

*Toxocara canis* eggs are highly resilient due to their robust structure, which consists of five distinct layers that provide exceptional protection against environmental adversity. This resilience facilitates egg survival and larval development (embryogenesis) under different environmental conditions [31]. The resilience of these eggs is partly responsible for the wide spread of infections in animals and humans [32,33,34,35].

Several approaches have been investigated to help control *T. canis* eggs in the environment, leading to significant scientific advancements. These approaches include chemical methods [36,37,38,39,40] and biological control agents [41,42,43]. Although chemical methods are effective, they can cause adverse environmental impacts, such as soil and water contamination, and may lead to parasitic resistance. Biological control agents, on the other hand, present a more environmentally friendly alternative. Nanobiotechnology approaches, especially those utilizing nematophagous fungi (green synthesis), such as *D. flagrans*, offer a promising alternative. However, further studies are necessary to fully understand the effectiveness of silver nanoparticles derived from *D. flagrans* against nematode eggs. In light of the foregoing, the aim of the current study was to evaluate the potential ovicidal activity of AgNP from *D. flagrans* on *T. canis* eggs.

## 2. Materials and Methods

### 2.1. Collecting T. canis Eggs

The eggs of *T. canis* were collected by dissecting the uterus of adult female nematodes obtained directly from the feces of naturally infected puppies [43]. After collection, the eggs were stored in a sterile Falcon tube with sterile distilled water to prepare aliquots and determine the egg concentration in the solution. The isolated eggs were then used in two experimental tests, namely Assay A, in which the ovicidal activity of AgNPs–*D. flagrans* on *T. canis* eggs was tested at different time intervals; and Assay B, which assessed the embryogenesis (larval development) of *T. canis* eggs over a 30-day period.

### 2.2. Producing D. flagrans Fungal Filtrate

In the current study, the isolate *D. flagrans* (AC001) from the mycology collection of the Laboratório de Parasitologia at the Department of Veterinary Medicine of the Universidade Federal de Viçosa, Minas Gerais, Brazil, was used and maintained at the Laboratório de Parasitologia Experimental e Controle Biológico, Universidade Vila Velha, Espírito Santo, Brazil. This isolate was continuously subcultured on potato dextrose agar (PDA, Kasvi^®^, Pinhais, Brazil).

*D. flagrans* was subcultured on Petri dishes covered with 2% chitin agar medium (AQ) consisting of agar-agar (Êxodo Científica^®^, Sumaré, Brazil) with 2% chitin (sterile tick shells) for the biosynthesis of AgNPs. The plates were incubated at 27 °C for 10 days. At the end of the incubation period, fungal discs (approximately 5 mm in diameter) were excised from the AQ cultures and inoculated into 250 mL Erlenmeyer flasks filled with 200 mL of low nutrient medium (LMN). The following components were used to prepare 1 L of LMN: 1 L ultrapure water (Milli-Q^®^, São Paulo, Brazil), 2 g bacteriological peptone, 3 g yeast extract, 0.1 g potassium dihydrogen phosphate, 0.05 g magnesium sulfate heptahydrate, 100 μL lactic acid, and 0.1 g chitin; the pH was adjusted to 9. The flasks containing the LMN and the fungal inoculum were incubated for 10 days at 120 rpm and 25 °C in an orbital shaker [15].

**Figure 1:**
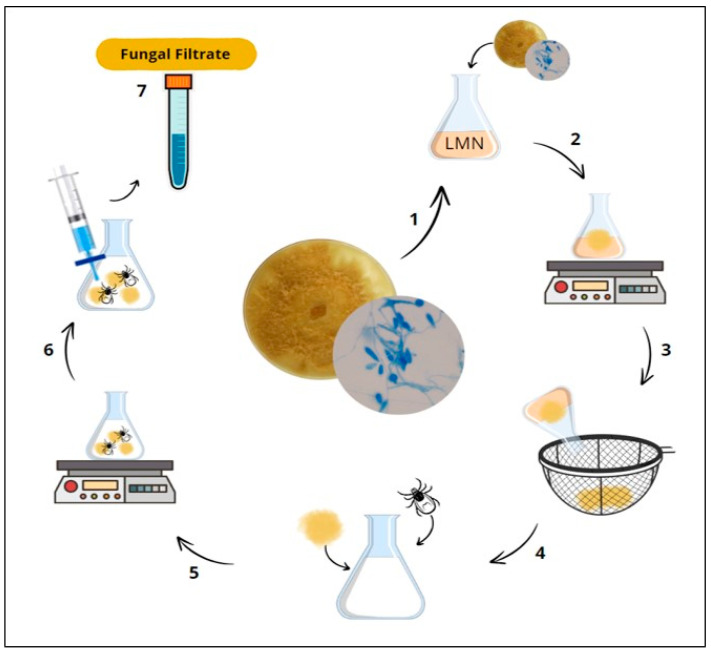
Schematic representation showing the preparation of fungal filtrate deriving from *Duddingtonia flagrans*. (1) The AC001 isolate was extracted from cultures grown on 2% AQ and inoculated in LMN; (2) Inoculated LMN incubation; (3/4) Fungal biomass formation and chitin addition; (5) Further incubation; (6/7) Culture contents’ filtration and fungal filtrate collection.

After 10 days of fungal growth in LMN, the fungal biomass was filtered and washed in ultrapure water. Subsequently, 100 mL ultrapure water, 10 g fungal biomass, and 0.1 g chitin were added to new 250 mL Erlenmeyer flasks, which were again incubated for 15 days at 120 rpm and 25 °C in an orbital shaker. The contents resulting from the incubation were filtered using syringes with 0.22 µm membrane filters to obtain the fungal filtrate (Figure 1).

### 2.3. Fungal Filtrate Protein Analysis

After the fungal filtrate was prepared, the total protein content was determined using the Bradford method [44]. Bovine serum albumin (BSA, Sigma^®^, São Paulo, Brazil) was used to establish the standard curve. Both the standard curve and the sample measurements were carried out in triplicate in the spectrophotometer at 595 nm.

### 2.4. Biosynthesis of Silver Nanoparticles (AgNPs) Derived from Fungal Filtrate

A 1 mM silver nitrate (AgNO_3_) solution was first prepared by dissolving 0.16987 g of AgNO_3_ in 1 L of ultrapure water to synthesize AgNPs. Then, the AgNO_3_ solution was mixed with the fungal filtrate in Erlenmeyer flasks at a ratio of 1:50 [22]. The flasks were incubated in an orbital shaker at 120 rpm, 60 °C, in the dark for 24 h.

### 2.5. AgNPs’ Biosynthesis Confirmation and Analysis

The biosynthesis of AgNPs was confirmed after a 24 h interaction of the fungal filtrate and the AgNO_3_ solution by measuring the absorbance rate using UV–Vis spectroscopy (Spectramax 190 spectrophotometer, San Jose, CA, USA) at a resolution of 1 nm and a scanning range of 200 to 600 nm. The control analysis was performed using the filtrate alone and excluding the AgNO_3_.

Subsequently, the solutions containing AgNPs were centrifuged at 12,000 rpm, 15 °C, for 20 min. Then, the resulting pellets were dried and resuspended in ultrapure water to reach the concentration of 43.40 μg/mL [22].

The samples were subjected to transmission electron microscopy (TEM, JEOL USA^®^, Peabody, MA, USA) to assess the morphology of the AgNPs. The diameter and distribution of AgNPs were analyzed using ImageJ version 1.5 software [45].

Dynamic light scattering (DLS) and zeta potential (ZP) measurements were performed to characterize the AgNPs–D*. flagrans*. DLS was used to determine the average size of the NPs in solution, while zeta potential analysis was performed to determine their surface charge. These analyses were conducted using a Zetatrac instrument, model NPA152.

A portion of the AgNP solution was centrifuged at 15,000 rpm for 30 min to form a pellet. This pellet was then analyzed by Fourier transform infrared spectroscopy (FTIR). FTIR analysis was performed in ATR mode (FTMIR FTLA 2000 Bomem, Sao Paulo, Brazil), according to the manufacturer’s guidelines, to identify the organic compounds associated with the AgNPs. The spectral resolution was set to 4.0 cm^−1^, and the wavenumber range was measured from 1700 cm^−1^ to 1200 cm^−1^. The resulting spectra were processed and analyzed using OriginPro version 9.4 software.

### 2.6. Assay A

**Figure 2:**
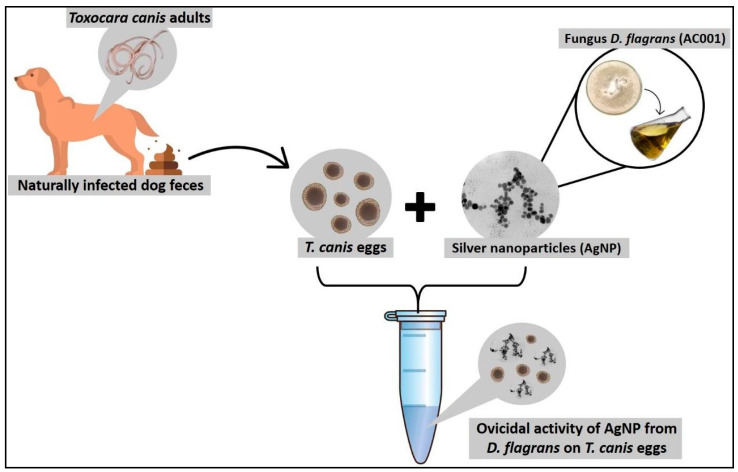
Overview of the adopted methodology. *Toxocara canis* eggs collected from fresh feces of naturally infected dogs were introduced in microtubes filled with AgNPs synthesized from *D. flagrans* fungal filtrate.

The following two experimental groups were formed: Group 1 (G1), or treatment group, consisted of 500 μL AgNPs–*D. flagrans* solution and 250 μL solution containing approximately 1300 *T. canis* eggs (Figure 2); whereas Group 2 (G2), or control group, consisted of 500 μL distilled water and 250 μL solution containing approximately 1300 *T. canis* eggs. Each group was subjected to two experimental periods—15 days and 30 days. Five replicates were performed for each experimental condition, and the final concentration of AgNPs–*D. flagrans* in G1, where the eggs were exposed, was 28.93 µg/mL.

The experimental setups were prepared in sterile 2 mL microtubes and kept at room temperature (25 °C ± 2 °C). The eggs of both groups were subjected to morphological analysis under the Nikon Eclipse Si microscope at the end of the 15- and 30-day periods. The following parameters were evaluated according to the modified methods [46,47,48]: (1) Shell integrity: intact or damaged/broken; (2) internal content: presence of embryo or degeneration; (3) coloration: translucent or dark/opaque; and (4) larval motility.

### 2.7. Assay B

The following two experimental groups were formed in sterile microtubes: Group 1 (G1), or treatment group, consisted of 120 μL AgNPs–*D. flagrans* solution and 60 μL solution containing approximately 300 *T. canis* eggs; while Group 2 (G2), or control group, consisted of 120 μL distilled water and 60 μL solution containing approximately 300 eggs. Five replicates were performed for each experimental condition, and the final concentration of AgNPs–*D. flagrans* in G1, where the eggs were exposed, was 28.93 µg/mL.

At the end of the 30-day incubation period, 100 *T. canis* eggs from each group were selected and analyzed under a light microscope to assess embryonic development and, more specifically, their complete larval stage. Only fully embryonated eggs with visible larvae were included in the statistical analysis. The viability of the larvae in the eggs was estimated based on the method described by Zhang et al. [48].

### 2.8. Statistical Analysis

The results observed for groups G1 and G2 in Assay A were subjected to an analysis of variance (ANOVA), followed by Tukey’s post hoc test at 5% significance level. All statistical analyses were performed using BioStat 5.0 software [49]. The following formula was used to determine the percentage reduction in the number of viable eggs [50]:

Reduction = (mean number of viable eggs in G2—mean number of viable eggs in G1)/(mean number of viable eggs in G1) × 100.

The killing efficacy of the treatment was calculated in Assay B based on the following formula [48]:

Killing effectiveness = (total number of eggs detected through microscopy—number of living eggs detected through microscopy)/(total number of eggs detected through microscopy) × 100.

## 3. Results and Discussion

### 3.1. Fungal Filtrate Production from D. flagrans and Protein Analysis

The total protein concentration in the fungal filtrate of *D. flagrans* was determined using the Bradford method (0.70 mg/mL). The protein content includes several hydrolytic enzymes produced by *D. flagrans*, such as proteases, pectinases, phospholipases and especially chitinase, as this fungus was grown in a medium enriched with chitin [15]. It is known that microorganisms can significantly increase their enzymatic performance under optimized culture conditions. Both biotic and abiotic factors can influence the productivity of these enzymes [51,52].

### 3.2. Silver Nanoparticles Biosynthesis

Within 24 h after the addition of AgNO_3_, in the initial phase of AgNPs biosynthesis, a clear color change from colorless to yellow occurred in the solution. This color change indicates a reduction of the silver ions (Ag⁺) to metallic silver, which led to the formation of the NPs [53]. The filtrate of *D. flagrans* facilitated this reduction process in this study.

UV–Vis spectroscopy was used to confirm the biosynthesis of AgNPs. UV–Vis spectroscopy of the AgNPs showed a clear peak at 414 nm, while no such peak was observed for the *D. flagrans* filtrate alone. This finding indicates that the observed peak is due to the presence of AgNPs. The wavelength of 414 nm is consistent with the characteristic absorption range of AgNPs, which typically show a peak between 380 nm and 450 nm [54].

**Figure 3:**
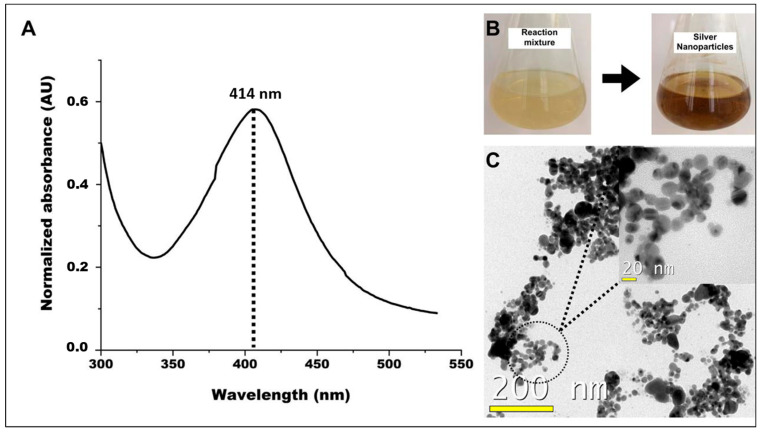
(**A**) The UV–Vis spectrum of AgNPs presented distinct peak at 414 nm. (**B**) Color change from colorless to yellow. (**C**) TEM-generated image showing AgNPs biosynthesized from *D. flagrans* fungal filtrate.

The AgNPs were subjected to morphological analysis using transmission electron microscopy (TEM) and analyzed in ImageJ2 version 1.5 software. The TEM images show that the AgNPs have a predominantly spherical shape and an average diameter of approximately 10.3 ± 7.2 nm. These observations confirmed the effective reduction of silver ions as well as the successful stabilization of AgNPs from the filtrate of *D. flagrans* (Figure 3).

To characterize an NP, it is crucial to determine its hydrodynamic size and surface charge in nanosuspensions. DLS and ZP measurements are commonly used to obtain this information [55]. The zeta potential of AgNPs–*D. flagrans* was measured at −35 mV. Suspensions with ZP values greater than +30 mV or less than −30 mV are considered stable as they indicate strong electrostatic repulsion between particles, which minimizes aggregation and precipitation thus ensuring high colloidal stability. In contrast, ZP values between −30 mV and +30 mV suggest instability [56,57]. Therefore, the AgNPs–*D. flagrans* solution with a surface charge of −35 mV was classified as stable.

**Figure 4:**
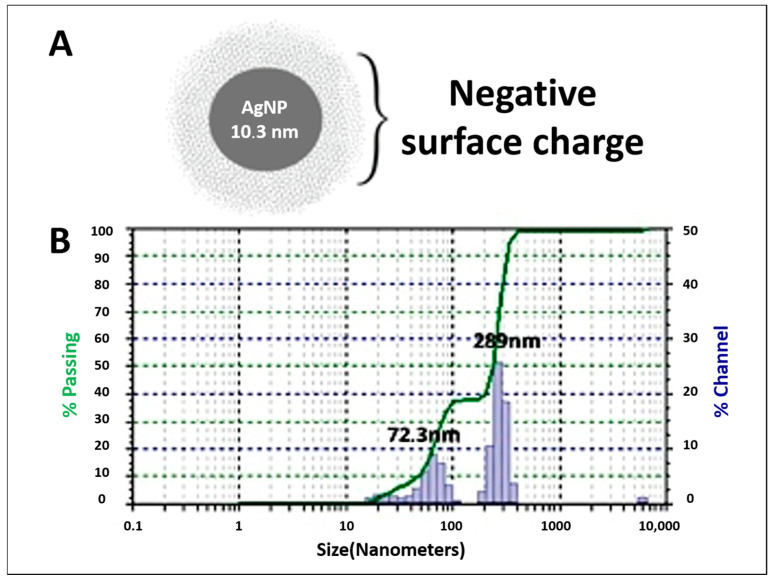
(**A**) Schematic representation of the average size of AgNPs–*D. flagrans* obtained from TEM and the negative charge determined by ZP measurements. (**B**) Hydrodynamic size of AgNPs obtained from DLS.

Figure 4A illustrates the average size of AgNPs based on TEM data together with their negative charge from ZP measurements. Using DLS, the predominant sizes of the biosynthesized AgNPs were determined to be 289 nm and 72.3 nm (Figure 4B). The size differences between TEM and DLS can be attributed to the distinct methods as follows: TEM provides a direct visualization of the metallic core of the nanoparticles, allowing for the precise measurement of their actual metallic size. In contrast, DLS evaluates the mobility of the nanoparticles in solution, which includes not only the metallic core but also any biological coatings or stabilizing substances on the surface of the nanoparticles, resulting in a larger measured size [58].

**Figure 5:**
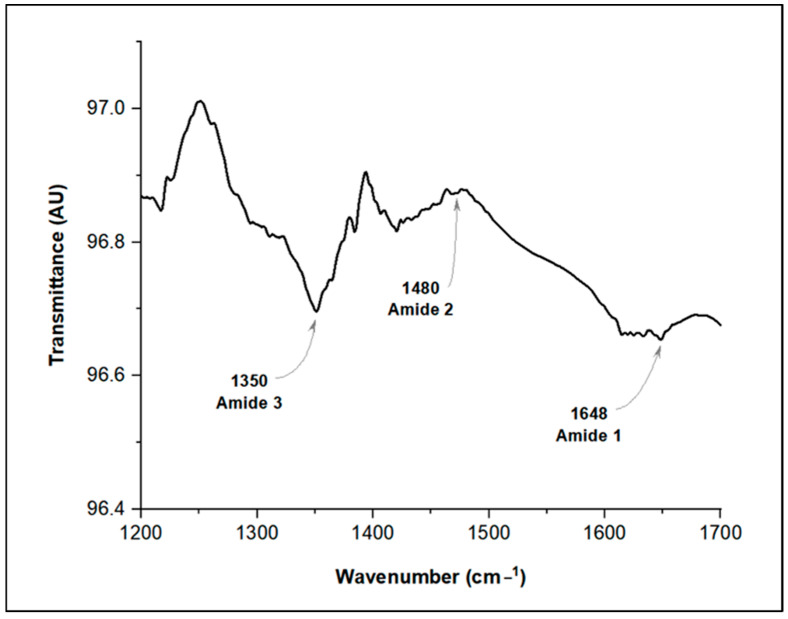
FTIR spectrum of the biosynthesized AgNPs–*D. flagrans*, showing the amide I, amide II and amide III bands.

To investigate the possible presence of chitinase as a biological coating on AgNPs–*D. flagrans*, an FTIR analysis was performed (Figure 5). Enzymatic structures, such as chitinases, contain peptide bonds characterized by amide bonds [59]. In this study, amide bands were identified in different spectral regions, providing insights into the organic compounds associated with the AgNPs.

The amide I band, the most prominent feature in the infrared spectrum, is particularly sensitive to the secondary structure of proteins and arises from the C=O stretching vibration of the amide group [60]. These vibrations typically occur between 1700 and 1600 cm^−1^; in the present work, a peak at 1648 cm^−1^ was observed [61]. The amide II band was detected at 1480 cm^−1^, indicating primarily N-H bending vibrations and C-N stretching vibrations [62]. Finally, the amide III band was identified at 1350 cm^−1^, which is a combination of C-N stretching and N-H bending vibrations. These results suggest the presence of amino acids, likely derived from chitinase, on the surface of the AgNPs–*D. flagrans*.

### 3.3. Nanoparticles Versus T. canis eggs

**Figure 6:**
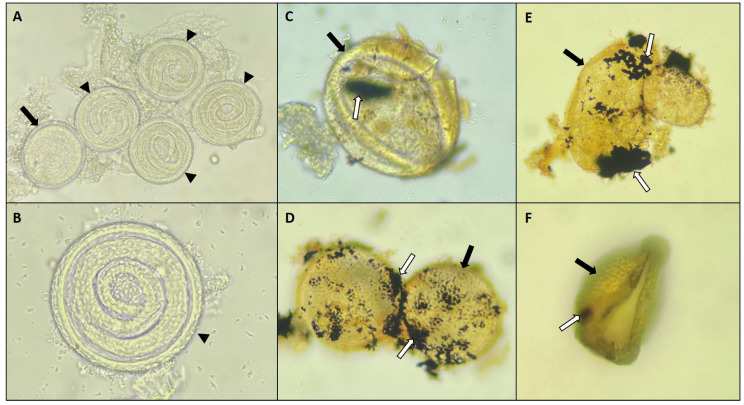
(**A**,**B**) Control group showing embryonated (black arrowhead) and non-embryonated (black arrow) *T. canis* eggs. (**C**–**F**) Non-embryonated *T. canis* eggs (black arrow) and eggs destroyed by silver nanoparticles deriving from *D. flagrans* fungal filtrate (white arrow).

The results of Assay A indicate that AgNPs derived from *D. flagrans* had a significant destructive effect on *T. canis* eggs in the treatment group compared to the control group (*p* < 0.05) (Figure 6, Table 1). By days 15 and 30 of treatment, 47% and 37% of eggs had been destroyed, respectively. Remarkably, the current study is the first to report the effects of AgNPs–*D. flagrans* on helminth eggs, as no previous research has documented such effects.

Light microscopy demonstrated significant damage in *T. canis* eggs belonging to the treated group (G1), such as the loss of shell integrity, with visible cracks and fractures, and the degeneration and leakage of internal contents. In addition, the eggs showed a clear color change from translucent to yellowish. In the study by Verocai et al. [46], the chemical effect of certain disinfectants on *T. canis* eggs was observed, showing changes in the structure and coloration of the eggs. Similarly, Zhang et al. [48] demonstrated that the chemical activity of disinfectants is responsible for structural changes and the inviability of the eggs. Therefore, in the present study, the same parameters (eggshell integrity, coloration, and larval motility) previously mentioned by these authors were used to evaluate the viability of *T. canis* eggs after exposure to AgNPs–*D. flagrans*.

The results in Assay B showed that 88% of the eggs in the group (G1) treated with AgNPs–*D. flagrans* did not develop to the larval stage, based on a sample of 100 eggs. In contrast, 68% of the eggs in the control group (G2) did not develop into embryos. This result shows that the rate of non-embryonated eggs in G1 was 29.4% higher than in G2.

However, it should be emphasized that although a considerable number of eggs in both groups did not embryonate, the G2 eggs showed no signs of physical destruction. The eggs in the control group kept their shells intact, with a transparent appearance and no leakage of the contents. On the other hand, the non-embryonated eggs in G1 showed visible signs of damage, such as shell breakage and leakage of the contents, indicating the destructive effect of AgNPs–*D. flagrans* on these eggs.

In addition, it is important to emphasize that the remaining 12% of eggs in G1 that were embryonated did not necessarily develop fully. According to Morgan-Jones et al. [63], the actual viability of these eggs and their embryos can only be definitively established through subsequent infection studies in animal models by choosing an observation period of more than 30 days.

*Toxocara canis* has infected over 100 million dogs and 1 billion humans worldwide. Embryonated *T. canis* eggs play a key role in both the transmission and prevalence of this zoonosis [64,65,66,67]. Therefore, the current study emphasizes the efficacy of AgNPs–*D. flagrans* in inhibiting the development of *T. canis* eggs into larvae.

Recently, significant progress has been made in research focused on investigating the biological activity of *D. flagrans* [17,68,69,70,71,72,73]. Biotechnological advances have shown that *D. flagrans*, among other nematophagous fungi, can produce both extracellular enzymes and silver nanoparticles, which in turn exhibit remarkable biological activity [15,17]. These findings highlight the potential of integrating fungal-based nanobiotechnology into biological control strategies applied to parasitic diseases.

The chitinases produced by *D. flagrans* play an important role in the reduction and stabilization of AgNPs. Fourier transform infrared (FTIR) and Raman scattering analyses have confirmed that chitinase is the key molecule responsible for both the reduction and stabilization of AgNPs, as evidenced by the presence of amino acids derived from this enzyme on the surface of the nanoparticles [15]. These findings are relevant and support the results of the current study, as chitin was used to produce AgNPs. In addition, FTIR shows the characteristic amide bands associated with enzymes (Figure 5). On the other hand, this result is in line with the reports of Braga et al. [17], who observed the production of chitinases by *D. flagrans* and emphasized the important role of these enzymes in infection processes and nematicidal activity.

The culture medium used in the current study not only induced chitinase production, but also indicated a dual role in AgNP synthesis and ovicidal activity against *T. canis* eggs. Although these results are still preliminary, they provide valuable insights into the nanoactivity of *D. flagrans* and may guide future research in this field.

The exact role of *D. flagrans* and its potential applications in the control of animal parasites were poorly understood for several years [73,74]. However, advances in scientific research have clarified its usefulness, and commercial products containing *D. flagrans* are currently widely used in animal husbandry for parasite control [17,20]. Barbosa et al. [22] determined the safe dose of 43.4 µg/mL for AgNPs–*D. flagrans* based on cytotoxicity tests. The aim of the current study was to build on the previous knowledge and explore the new discoveries and future applications of AgNPs–*D. flagrans* to further improve both the understanding and potential use of this fungal strain to control parasitic infections.

The process to control *T. canis* and other nematodes has been hampered for decades by therapeutic failures, largely due to the growing population of stray dogs and cats in urban areas [26,53,63]. Therefore, research into new biological control strategies in the environment may help to find promising solutions for the effective control of these nematodes. Barbosa et al. [22] and Ferraz et al. [23,24] have already shown that AgNPs–*D. flagrans* exhibit nematicidal activity against nematode larvae. The present study extends these findings by showing that these AgNPs also have ovicidal effects on these nematodes. Nevertheless, it is important to conduct further studies in vivo to improve the knowledge on this topic.

## 4. Conclusions

AgNPs–*D. flagrans* were found to be effective in destroying *T. canis* eggs, significantly impairing their integrity and inhibiting their development. Furthermore, TEM, DLS, ZP, and FTIR techniques were used to evaluate the morphological characteristics and stability of AgNPs. Therefore, these results can be used in future studies to investigate the ovicidal potential of these biomolecules.

## Figures and Tables

**Table 1:** Means and standard deviations recorded for AgNPs–*D*. *flagrans* ovicidal activity in *Toxocara canis* eggs (G1) in comparison to the control group (G2) at days 15 and 30.

Days	G1	G2	%R (Ovicidal)
15	131.4 ^a^ ± 46.2	249.1 ^b^ ± 45.8	47%
30	116.6 ^a^ ± 45.3	185.1 ^b^ ± 59.2	37%

Means followed by a different letter in the lines mean (*p* < 0.05)—Tukey test.

## Data Availability

The original contributions presented in this study are included in the article. Further inquiries can be directed to the corresponding author.
